# Nanoscale Topographical Effects on the Adsorption Behavior of Bone Morphogenetic Protein-2 on Graphite

**DOI:** 10.3390/ijms23052432

**Published:** 2022-02-23

**Authors:** Izabele Marquetti, Salil Desai

**Affiliations:** 1 Department of Biomedical Affairs, Edward Via College of Osteopathic Medicine, Spartanburg, SC 29303, USA; ibmarquetti@gmail.com; 2 Center for Excellence in Product Design and Advanced Manufacturing, North Carolina A&T State University, Greensboro, NC 27411, USA

**Keywords:** adsorption, bone morphogenetic protein-2, graphite, molecular dynamics, nanoscale topographies, regenerative medicine, tissue engineering

## Abstract

The interaction between bone morphogenetic protein-2 (BMP-2) and the surface of biomaterials is essential for the restoration of bone and cartilage tissue, inducing cellular differentiation and proliferation. The properties of the surface, including topology features, regulate the conformation and bioactivity of the protein. In this research, we investigated the influence of nanopatterned surfaces on the interaction of a homodimer BMP-2 with graphite material by combining molecular dynamics (MD) and steered molecular dynamics (SMD) simulations. The graphite substrates were patterned as flat, linear grating, square, and circular profiles in combination with BMP-2 conformation in the side-on configuration. Ramachandran plots for the wrist and knuckle epitopes indicated no steric hindrances and provided binding sites to type I and type II receptors. Results showed two optimal patterns that increased protein adsorption of the lower monomer while preserving the secondary structure and leaving the upper monomer free to interact with the cells. Charged residues arginine and lysine and polar residues histidine and tyrosine were the main residues responsible for the strong interaction with the graphite surface. This research provides new molecular-level insights to further understand the mechanisms underlying protein adsorption on nanoscale patterned substrates.

## 1. Introduction

The interfacial phenomenon directly affects the effectiveness of biomedical devices. Surface properties of biomaterials guide essential in vivo functions, including protein adsorption, cell adhesion, and inflammatory responses [1]. The modification and functionalization of biomaterials allow the development of an adequate physical and chemical environment, inducing specific cellular functions and stimuli-responsive release [1,2]. Surface patterning includes topological or morphological features on the surface of the biomaterial that facilitate the adhesion, proliferation, and migration of cells. In biological systems, the three-dimensional structure of the extracellular matrix provides specific signals that induce cellular development [2,3].

Signals and biomaterials play an important biological role in the enhancement of cell therapy [4,5]. However, one of the critical issues in tissue engineering-based treatment is to obtain an efficient delivery of the proteins to the injury site [6,7,8]. Usually, this delivery has a weak tissue penetration, an uncontrollable migration, and incompetent internalization of cells due to the enzymatic degradation of the extracellular matrix [9]. When the biomaterial is inefficient, the growth factors are not protected and get exposed to inflammatory conditions, limiting their activity [10]. Further, biomaterials may need supporting coatings or delivery mechanisms to sustain their bioactivity [11,12,13]. Therefore, understanding the interaction of these signals with biomaterials is essential to improve cell growth.

Patterned surfaces present a challenge to protein adsorption for biomedical applications because these topographies include regions that can either promote affinity/adhesion of proteins or have significant resistance to protein adsorption [3,14]. Different surface topographies have been studied to control cell morphology, including grooves and ridges, wherein the pattern dimensions directly affected cellular growth [15,16], which are old references. However, limited studies investigated the influence of specific patterned surfaces on protein adsorption and bioactivity [17,18,19]. The presence of topographical patterns of biomaterials is essential to obtain favorable protein-surface interactions for applications in tissue engineering. Molecular dynamics (MD) simulation is an efficient method to investigate protein adsorption processes, allowing molecular-level characterization of the conformational changes occurring in the protein structure during adsorption [20,21].

Bone morphogenetic proteins (BMPs) are an essential type of signal belonging to the transforming growth factors beta (TGF-β) superfamily. BMPs are directly involved in many developmental processes for tissue and organ restoration [22,23]. Although BMPs have some similarities with the other proteins of the TGF-β superfamily, they present more complex signaling functions, which makes their comprehension essential to tissue restoration. BMPs are usually separated into four subgroups according to their sequence similarities and functions: BMP-2/4, BMP-5/6/7/8a/8b, BMP-9/10, and BMP-12/13/14 [23]. BMP-2 is one of the most important proteins for the formation and restoration of bone structure due to its ability to promote the differentiation and proliferation of osteoblasts. Along with standard procedures for bone repair, BMP-2 has proven to reduce the time of healing and the risk of rejection, allowing savings in the treatment cost. However, its application for clinical use faces some challenges regarding its delivery system due to uncontrolled release from the application site and undesirable effects at distant sites [24,25].

The effects of surface patterning on the behavior of BMP-2 still lack understanding, as the spacing and size of the patterns can be an important tool for controlling the quantity of immobilized proteins at the molecular level [25]. Currently, only a few studies have investigated the influence of patterned surfaces on BMP-2 adsorption using molecular dynamics simulations. Huang et al. [26] studied the interaction of homodimer BMP-2 on nanostructured hydroxyapatite, concluding that increased surface roughness similar to HAP crystal polished surface (thickness 33.1 ± 5.6 nm) can promote enhanced protein-surface binding. Huang et al. [27] investigated the adsorption and desorption behavior of BMP-2 on different nano-texturized hydroxyapatite structures, obtaining fewer conformation changes of the protein molecule and higher stability of cysteine-knot of texturized HAP-1:1 (ridge:groove ratio) as compared to a flat substrate. Mucksch et al. used an implicit inviscid water model to study the adsorption dynamics and energetics of four different initial BMP-2 orientations on a graphite substrate [28]. The unfolding of a BMP-2 molecule was observed, leading to denaturation of the protein structure. However, a monomer BMP-2 model was used, and thus, the results obtained cannot be applicable as a bioactive form of BMP-2 exists in the dimer format for biomedical usage. Accelerated molecular dynamics was used to study graphite in an explicit saltwater environment [29]. A comparison between classical and accelerated MD was used to show differences in the sampling of conformational changes during adsorption on a hydrophobic graphite surface. Copolymer micellar nanolithography was used to fabricate substrate tunable gold nanoparticle arrays carrying single BMP-2 molecules [25]. It was revealed that immobilized growth factors triggered an elevated Smad signaling pathway activation compared to soluble protein.

In our earlier investigations, we explored the adsorption behavior of four orthogonal orientations of BMP-2 protein with hydrophilic silicon dioxide substrate [30]. Our findings suggest that the end-on configurations had favorable adsorption characteristics along with a robust secondary structure. The BMP-2 adsorption was studied with hydrophobic (gold) and hydrophilic (silicon nitride) flat surfaces [31]. The interaction of BMP-2 with silicon nitride substrate resulted in denaturation of the secondary structure into coil formation. However, gold substrate offered structural stability and higher adsorption. BMP-2 interaction with substrate is mediated between the protein, the dissolution media, and the substrate. Of all the configurations, the end-on configuration provided the highest adsorption characteristics for all the substrates and was, thus, deemed suitable for consideration. Our recent investigations with a nanopatterned gold substrate with deeper grooves showed higher adsorption behavior and retention of the secondary structure due to binding at the bottom and sidewalls of the grooves [32]. In the graphite substrate, we studied flat substrates based on their higher biocompatibility, low friction coefficient, and bone equivalent stiffness before investigating nanopatterned graphite substrates [33]. There are few studies that consider molecular investigations of BMP-2 on graphite substrate, and thus, a comprehensive understanding of nanoscale patterning on the BMP-2 adsorption is warranted.

In this research, MD simulations were used to investigate the influence of different nanoscale topographical patterns of graphite on the protein adsorption of bone morphogenetic protein-2 (BMP-2). BMP-2 is an important protein for the formation and restoration of bone tissue, promoting the differentiation and proliferation of osteoblasts. Due to the increased biocompatibility, low friction coefficient, and right stiffness with bone, pyrolytic graphite is commonly used as an implant material. However, there are limited studies on the interaction of this material with osteogenic protein that can promote the differentiation of stem cells [34]. Herein lies the premise of this research, which aims to investigate the adsorption mechanism of BMP-2 on a nanoscale patterned graphite substrate using computational tools to provide guidance for in-field implementation.

## 2. Results and Discussion

**Figure 1:**
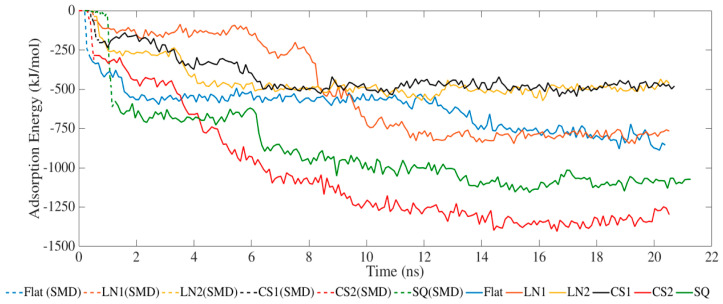
Adsorption energy between BMP-2 protein and substrate.

The adsorption process occurred exclusively via van der Waals interactions due to the lack of partial charges (Figure 1). SMD simulations allowed the protein to closely approach the surface, increasing the adsorption energies. The simulated systems had the same binding energy profile but with different intensities. LN1 had similar energies compared to a flat surface, which occurred due to the wide grating width of 90 Å. In the case of LN1, adsorption occurred exclusively on the bottom of the substrate. Therefore, the adsorption process had no influence on the pattern for LN1, behaving similarly to a flat surface. Increased adsorption was obtained with the systems simulated with CS2 and SQ. The values of adsorption energy significantly increased after SMD simulations, reaching −1423 and −1130 kJ/mol for CS2 and SQ, respectively. Both of these patterned surfaces maximized the number of residues contacting the substrate, therefore increasing the binding. Additional binding sites were located on the sidewalls of CS2 and SQ profiles, which contributed to the adsorption energies. However, relatively lower binding occurred for CS1 as the BMP-2 protein did not interact closely with the semi-hemispherical slot in the substrate. On the contrary, the CS2 circular slot had a conforming shape matching the BMP-2 molecule profile, which significantly increased adsorption behavior.

**Figure 2:**
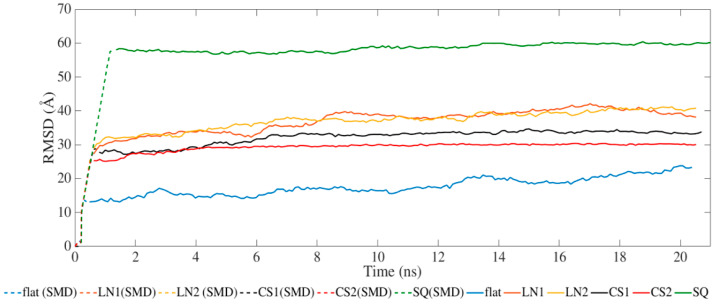
Root-mean-square deviation of BMP-2 over time.

Figure 2 presents the results for the average RMSD of the atomic coordinates throughout the MD and SMD simulations. All systems achieved equilibration during the SMD phase with steady values of RMSD during the relaxation phase. The different topographical patterns induced an increase in RMSD compared to the flat surface. Simulations performed on CS2 and SQ presented less fluctuation after SMD than the others.

BMP-2 promotes tissue restoration mainly through oligomerization of the receptor types I and II, transmembrane serine/threonine kinase receptors that form a receptor-ligand complex [35]. During the BMP signaling pathway, the homodimer binding promotes the development of hetero-tetrameric receptor complexes formed by BMPR-IA, BMPR-IB, and BMPR-II, activating phosphorylate Smad [36]. The wrist epitopes (residues Phe49 to Val63) and knuckle (residues Ala34, His39, Ser88, Leu90, and Leu100) are responsible for binding to type I and II receptors, respectively, allowing initiation of Smad pathways [37]. Therefore, it is essential for the homodimer to have its secondary structure of the wrist and knuckle epitopes preserved throughout the binding. To evaluate the protein conformation, we analyzed the secondary structure of the homodimer and the Ramachandran plot at the beginning and the end of the simulations.

**Figure 3:**
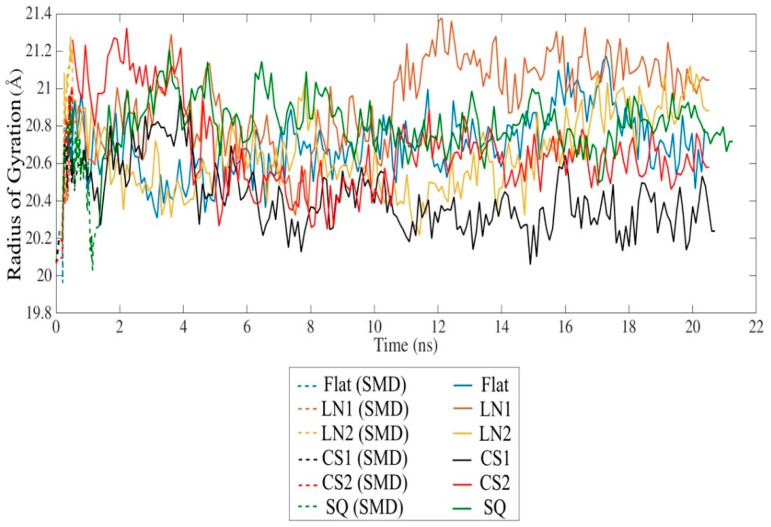
Radius of gyration of the BMP-2 homodimer over time.

SMD simulation effectively approached the protein towards the substrate surface without causing significant changes in the secondary structure. The radius of gyration indicates a slight unfolding of the protein throughout the simulation in all cases (Figure 3). However, the presence of circular slots prevented the unfolding behavior, as compared to the flat and linear gratings substrates. No significant changes of the secondary structure occurred after 20 ns relaxation, indicating that the protein remains bioactive even with strong adsorption on CS2 and SQ, with an average R_g_ of 20.67 Å and 20.73 Å, respectively. Changes in the secondary structure occurred mostly on the adsorbed monomer A, especially on β-sheets. A slight formation of α-helix occurred in almost all monomers, while 3_10_-helix was formed exclusively during simulations on SQ. LN1 presented a more unfolded structure than the others, which is consistent with the loss of β-sheet structures. Simulations performed on the flat substrate revealed that increased conformational changes occurred even with lower adsorption compared to the models CS2 and SQ, which had increased adsorption. The reduction of the β-sheet represents 8.5%, 14.0%, and 19.4% of the original structure for flat, CS2, and SQ, respectively, while only on the flat substrate a reduction of α-helix occurred (9.1%). Therefore, specific patterned surfaces can induce adsorption and, at the same time, preserve the secondary structure, which is vital for the proper interaction with the cells.

**Figure 4:**
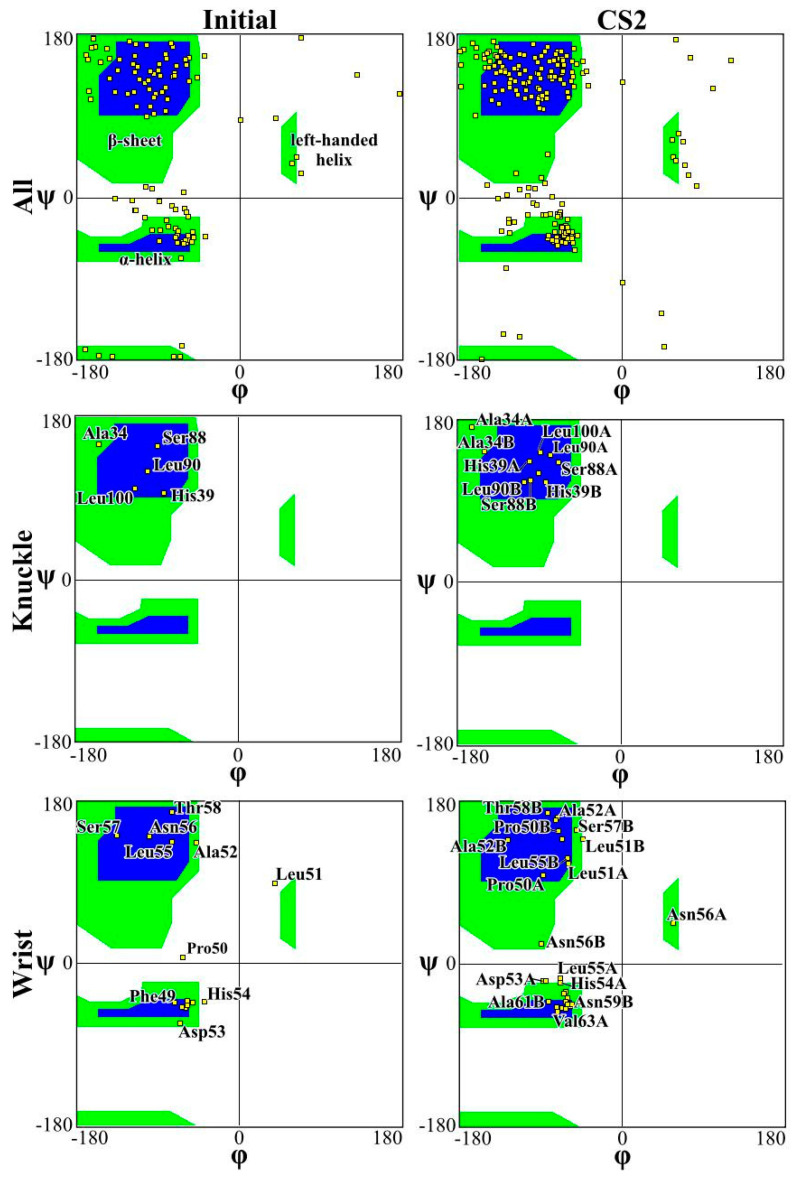
Ramachandran plot for analyzing 3D of the BMP-2 homodimer of CS2 configuration.

**Figure 5:**
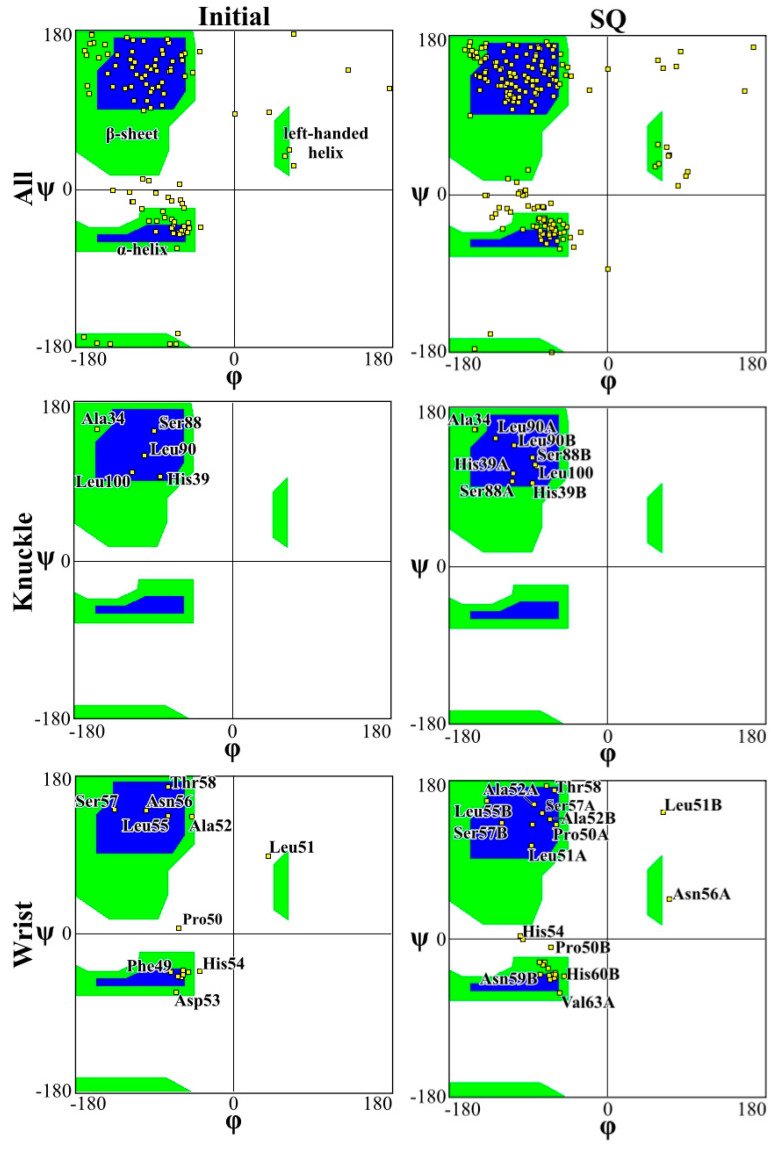
Ramachandran plot for analyzing 3D of the BMP-2 homodimer SQ configuration.

Figure 4 and Figure 5 present the Ramachandran plot for the simulations performed on CS2 with SQ and patterns at the beginning and end of the simulations, respectively. These plots demonstrate the conformation of the protein throughout the simulations. In a polypeptide chain, the torsion or dihedral angle of the N-C_a_ and C_a_-C bonds determines the three-dimensional structure of the protein backbone. The distribution of the torsion angles ψ (angle around C_a_-C bonds) and φ (angle around N-C_a_ bonds) of both monomers in the protein, showing all residues and those of the wrist and knuckle epitopes. In Figure 4 and Figure 5 the blue regions indicate the favorable areas for α-helix and β-sheet structures, the green regions are less favorable areas, and the white regions are disallowed regions where the loss of secondary structure occurs. The figures show that for the simulations performed with CS2 and SQ, both models present minimal changes of the wrist and knuckle epitopes, even though they had higher conformational changes of the β-sheet structures. Therefore, for CS2 and SQ configurations, the protein remains bioactive to promote tissue restoration.

**Figure 6:**
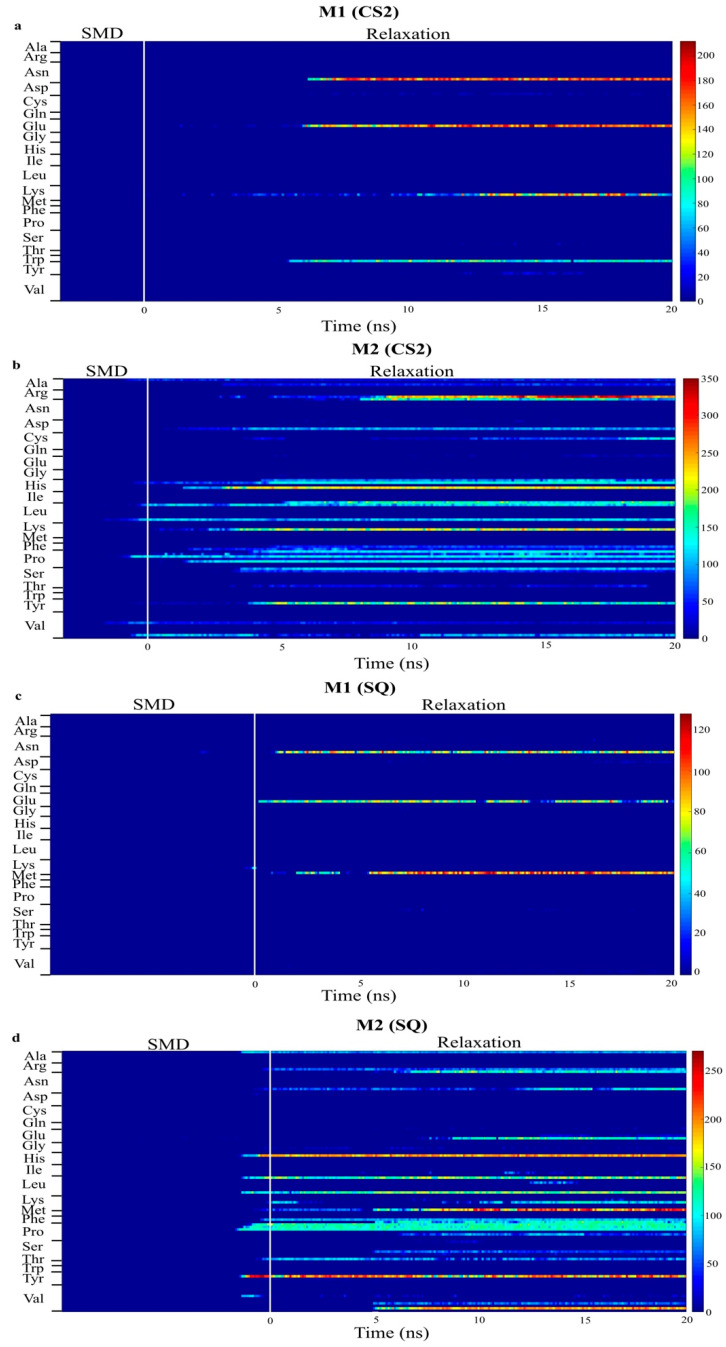
Number of contacts between atoms in each residue of monomer A and B within 5 Å of graphite surface for CS2 and SQ graphite substrates. ((**a**,**b**) are bottom and top monomer for CS2 profile, whereas (**c**,**d**) are bottom and top monomer for SQ profile, respectively.)

The Ramachandran plots for the wrist epitopes for both topographies show that residues are available to permit binding to type I and type II receptors, thereby initiating the SMAD-signaling pathway, which in turn regulates downstream biological processes. The CS2 and SQ substrate patterns displayed minimal steric hindrances for both the wrist and knuckle epitopes, indicating a stable and well-conformed secondary structure as validated by α-helix and β-sheet values shown in Table 1. In addition, a significant number of residues belonging to the wrist epitopes (Thr58, Leu55, Leu51, Ala52, Leu90, Leu100) and knuckle epitopes (His 54, Asp 53, Val 63, Asn59B, Val63A) in the CS2 and SQ topographies maintained their secondary structures providing binding sites for type I and II receptors. It is also important to note that several of these residues have formed intermediate and high-affinity binding sites with the graphite substrate, as explained in Figure 6. Thus, the Ramachandran plot clearly indicates favorable residues for both wrist and knuckle epitopes in both CS2 and SQ topographies that can promote successful adsorption and bioactivity.

Figure 6 presents the number of contacts of the residues within 5 Å for the models with increased adsorption after relaxation, CS2 and SQ. This plot quantifies the number of adsorbing atoms in each residue interacting with the surface of the substrate. The blue region represents no interaction between protein and surface, whereas the red region represents a higher number of atoms adsorbing. Intermediate interaction is represented by the yellow region. Limited interaction occurred between the monomer B and the substrate for both configurations, allowing the residues to be free to interact with the cells. Different residues were responsible for the adsorption process on these configurations. In the simulations performed on CS2, stronger protein-surface interaction occurred with the positively charged residues Lys15, with more than 200 atoms contacting the surface, and Arg16, with up to 350 atoms, and polar residue His54, with more than 200 atoms, of the monomer A, which occurred after SMD. Other residues in monomer A responsible for intermediate adsorption (100 to 200 atoms) included Arg9, Leu10, Ser12, Pro18, Tyr20, Pro36, His39, Pro50, Asp53, Leu90, and Leu100. Limited interaction occurred between monomer B and the surface; only residues Trp31, Lys73, Glu94, Asn95 adsorbed with less than 150 atoms. The residues Glu94 and Asn95 are part of a flexible loop of hydrophilic residues, facilitating the first approach to the surface during SMD.

In the simulations performed on SQ, the adsorption process was initiated at the end of SMD simulations. Four residues contributed to the stronger interaction of monomer A with the surface, polar residues Tyr20 and His39, with 200 atoms contacting the adsorption area, and hydrophilic residues Lys97 and Val98, with 230 and 200 atoms, respectively. Intermediate adsorption occurred with the residues Arg9, Lys15, Pro18, Pro35, Pro36, Phe49, Leu90, Glu96, and Leu100 of monomer A. For monomer B, residues Asn95, Glu96, and Lys97, which are also part of the flexible loop mentioned above, adsorbed with 70–100 atoms. In our previous study [34], in which we evaluated the effects of different initial orientations of a monomer BMP-2 onto graphite on the adsorption behavior, similar residues were responsible for adsorption on flat graphite, including Tyr, His, Arg, Val, and Lys. These findings show the strong influence of a specific group of residues on the adsorption behavior of BMP-2 on graphite.

**Figure 7:**
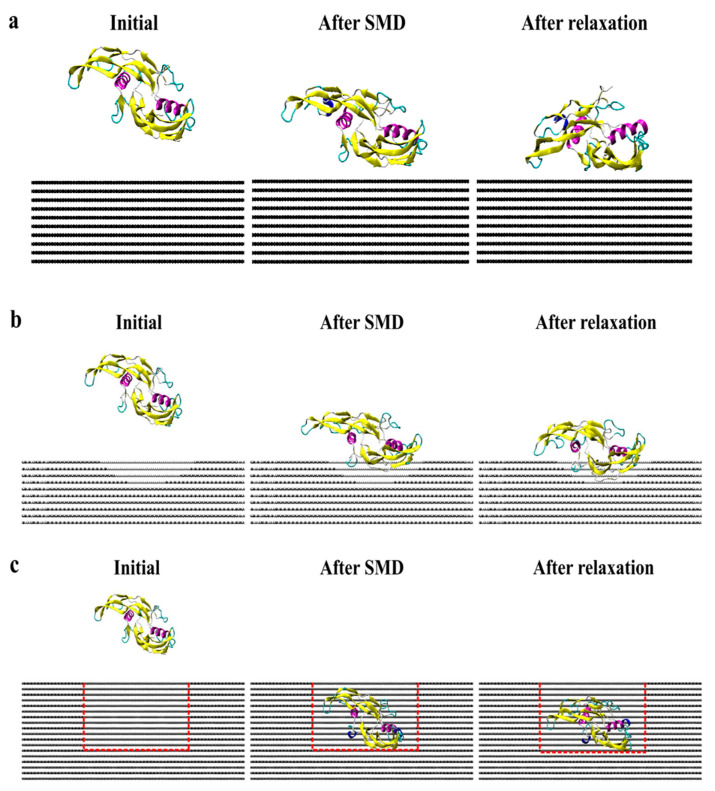
Side view of protein trajectory after SMD and 20 ns relaxation for (**a**) flat, (**b**) CS2, and (**c**) SQ graphite surfaces.

Figure 7 presents screenshots of the homodimer trajectory throughout the CS2 and SQ simulations. Minimal conformational changes occurred during CS2 simulation; monomer A achieved a stable structure since the early stages of relaxation. The CS2 pattern was able to increase protein adsorption while preserving the structure of the adsorbed monomer. According to Venkatakrishnan and Kuppa [38], protein-surface interaction is directly affected by the surface roughness, in which topologies with a similar format as the protein can promote increased adsorption because of the proximity of residues with the surface curvature, as compared to a smooth surface [38]. Although SQ promoted increased adsorption, the homodimer presented a more unfolded configuration than the simulations performed on CS2, which can explain the losses of the secondary structure. Thus, both CS2 and SQ topographical patterns provided both structural stability and adsorption sites, thereby ensuring higher bioavailability for tissue engineering applications.

**Figure 8:**
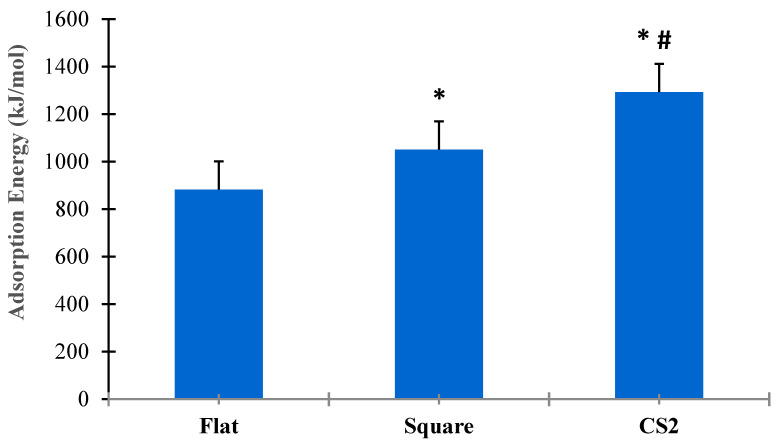
Comparative analysis of adsorption energies for flat, square, and CS2 graphite substrates (*p* < 0.05). * Indicates statistically significant difference between flat and square profile, * # indicates statistically significant difference between CS2 and flat, CS2 and square profile, respectively.

Figure 8 shows the statistical analysis for adsorption energies for flat, square, and CS2 graphite substrates at (α = 0.05). Three simulation runs (*n* = 3) were conducted for the entire duration of both SMD and relaxation cycles of the protein on each substrate. The *t*-test for paired sample means clearly show that there was a statistical difference among the adsorption energies between flat, square, and CS2 nanopatterns based on *p* < 0.05. The CS profile had the highest mean adsorption energy (1292.54 kJ/mol), followed by the square profile (1050.87 kJ/mol) and the baseline flat substrate (882.25 kJ/mol). Thus, it is evident that nanoscale topographies induce higher adsorption to the graphite substrate while maintaining their secondary structures and bioavailability. Moreover, nanoscale profiles that confirm the ternary and quaternary complexes of the BMP-2 molecule provided the highest adsorption energy as observed with the CS2 profile.

## 3. Methods

Combined steered molecular dynamic (SMD) and MD simulations were performed using NAMD source code version 2.11 [39] with CHARMM force field on a 64-bit Linux platform (Fedora 21). Two graphical processing units (GPUs) from NVIDIA^®^ Corporation, Santa Clara, California, USA, K20 and K40 (2496 and 2880 cores, respectively), were used to accelerate the simulations. Additionally, simulations were performed with GPU computing resources, K80 (4992 cores) provided by XSEDE [40]. GPUs have been successfully used to solve complex simulations in different areas, including biological systems [41].

**Figure 9:**
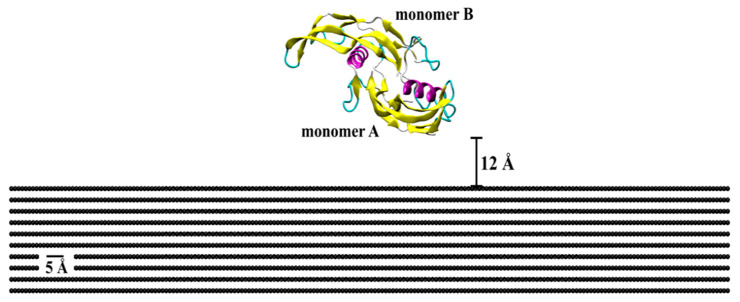
Initial crystallographic structure of BMP-2 homodimer placed on a flat graphite substrate. Protein is represented by the secondary structure, in which pink is α-helix structures, yellow is β-sheets, cyan is β-turn, and whitish-grey is coil structure.

The initial crystallographic structure of BMP-2 (ID: 3BMP) was taken from the RCSB Protein Data Bank (Figure 9) [42]. The bioactive form of this protein is a homodimer bonded by a disulfide bridge between residues Cys78 (cysteine) of each monomer (A and B). Monomer A is the lower among the two, with close proximity of the substrate, as compared to monomer B, which is free to attach with binding sites for SMAD pathway interactions. Each monomer contains one α-helix of four-turns and two anti-parallel double-stranded β-sheets. Lysine (Lys) and arginine (Arg) were protonated, while glutamic acid (Glu) and aspartic acid (Asp) deprotonated. All histidine (His) residues were considered as protonated state Hse.

The native-state protein was solved in bulk water with an explicit TIP3P model, and Na^+^ and Cl^−^ ions were added at 0.15 mol/L concentration using Visual Molecular Dynamics (VMD). The resulting model had 252,346 atoms and a total system charge of 2.28 × 10^−6^ (i.e., neutral). The bonded and non-bonded parameters of the water and the protein molecules were acquired from the CHARMM force fields [43]. The solvated system was placed on top of different graphite substrates, with the homodimer positioned in side-on orientations. This orientation has been proven to improve BMP-2 adsorption on different substrate materials in previous studies, including graphite [33], silicon dioxide [30], silicon nitride, and gold [31].

**Figure 10:**
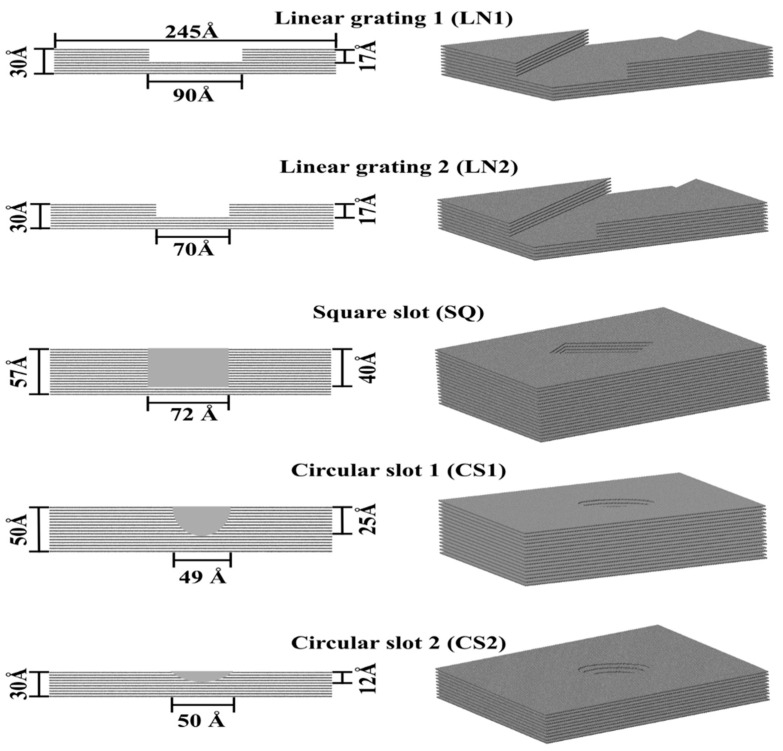
Topographically patterned surfaces of graphite.

Protein adsorption was evaluated on different patterned surfaces of graphite, including circular slots, linear grating, square slots, and compared to a flat substrate (Figure 10). All graphite substrates were created using a VMD plugin Inorganic Builder (unit cell parameters *a* = 1.228, *b* = 2.127, and *c* = 6.696), with a lattice plane (001). The flat substrate had dimensions of 245 × 423 × 30 Å. The patterned substrates were created by making an exclusion in a flat substrate. The solvated system was placed and centered on the top of the substrates with an average distance between the protein and top molecules of the substrate of 12 Å.

The non-bonded force field parameters for graphite were σ_ij_ = 3.195 Å and ε_ij_ = −0.439 kJ/mol [44], reproducing a contact angle of 86°. The atoms in the substrates were fixed to maintain computational efficiency. Periodic boundary conditions were considered in the *x*- and *y*-directions to avoid the effects of the edges of the simulation box on the protein adsorption. In the *z*-direction, a hard wall constraint was set on both the top and bottom of the box. All systems were minimized for 0.2 ns with a cutoff distance of 12 Å for VdW interactions. The temperature was maintained constant at 310 K using Langevin temperature control [45]. After minimization, SMD simulations of constant velocity pulling (k = 167.36 kJ mol^−1^Å^−2^, ν = 0.001 Å ps^−1^, and time according to Table 2) were performed, applying uniform forces on the backbone atoms of the protein. The SMD phase was followed by an MD relaxation phase conducted for 20 ns. The variation in the SMD time steps depended on the topographical feature and depth of the exclusion.

Protein adsorption was evaluated by the adsorption energy between the protein and substrate, which accounts for the non-bonded energy. The non-bonded energy corresponds to the van der Waals energy (Equation (1)), a weak attractive/repulsive force due to non-covalent interactions, and electrostatic energy (Equation (2)), due to different charge distributions.

(1) $$U_{\mathrm{Coulomb}}=\underset{i}{\sum }\underset{\;j\;>1}{\sum }\frac{q_{i}q_{j}}{4\pi \varepsilon_{0}r_{\mathrm{ij}}}$$

(2) $$U_{\mathrm{VdW}}=\underset{i}{\sum }\underset{\;j>1}{\sum }4\varepsilon_{\mathrm{ij}}\left[{\left(\frac{\sigma_{\mathrm{ij}}}{r_{\mathrm{ij}}}\;\right)}^{12}-\;{\left(\frac{\sigma_{\mathrm{ij}}}{r_{\mathrm{ij}}}\;\right)}^{6}\right]$$

where $r_{\mathrm{ij}}$ the distance between atoms sites i and j, q is the electric charge,$\;\varepsilon_{\mathrm{ij}}$ is the depth of the potential well, σ_ij_ is the distance where the potential is zero.

The root-mean-square deviation (RMSD) was quantified to obtain the position of the atoms during the MD and SMD simulations (Equation (3)). RMSD is also an indication of the equilibration of the system [16].

(3) $$\mathrm{RMSD}=\sqrt{\frac{\sum_{j=1}^{{\;N}_{t}}\;\sum_{i=1}^{N_{\alpha }}\;{\left(r_{i}\left(t_{j}\right)-{\;r}_{i}\left(t_{1}\right)\right)}^{2}}{N_{\alpha }}}$$

where, $N_{t}$ is the number of time steps, $N_{\alpha }$ is the number of atoms whose positions are being compared, and $r_{i}\left(t_{j}\right)$ is the position of atom i at time $t_{j}$. The adsorption process was investigated by the binding energy and the number of contacts made by the residues. The binding energy corresponds to the non-bonded protein-substrate interaction energy [37], which is the Van der Waals and electrostatic energies. The number of contacts corresponds to the number of atoms in a single residue within 5 Å of the substrate surface.

A significant folding/unfolding of the protein leads to loss of bioavailability and denaturation. Therefore, the level of folding/unfolding of the protein was evaluated by the radius of gyration (R_g_), characterizing the protein conformation (Equation (4)). Where: $\left|r_{i}{-r}_{\mathrm{com}}\right|$ is the distance of the atom i with mass $m_{i}$ to the center of the mass.

(4) $$R_{g}=\sqrt{\frac{\sum_{i}{\left|r_{i}-r_{\mathrm{com}}\right|}^{2}m_{i}}{\sum_{i}m_{i}}}$$

To analyze the protein bioactivity, the secondary structure of the initial and final configurations were compared. For the protein to be bioactive, no significant denaturation can occur, which happens when the α-helix and β-sheet structures are broken, and a random coil remains [46]. In addition, Ramachandran plots were used to analyze significant changes in the ψ and φ torsion angles of the residues, validating the secondary structure of the protein based on minimal steric hindrances during the adsorption phase.

## 4. Conclusions

In this study, we investigated the adsorption behavior of a BMP-2 homodimer on different nanopatterned graphite substrates combining molecular dynamics and steered molecular dynamics simulation. Our results indicate that specific patterns can influence the adsorption behavior of the protein. The CS2 and SQ substrates allowed increased adsorption compared to the flat surface, with low conformational changes of the molecules. The adsorption occurred mainly with the lower monomer (A) of the dimer, which would allow the other monomer (B) to interact with the cells. Protein-biomaterial interactions occurred exclusively via van der Waals energy due to the lack of partial charges of the substrate. Ramachandran plots indicated that minimal steric hindrances were observed for both wrist and knuckle epitopes. The secondary structures for α-helix and β-sheets were preserved, ensuring bioavailability for the SMAD-signaling pathway. Increased adsorption occurred along the sidewalls and profiled shape of the CS2 and SQ substrates without significant changes on the secondary structure of the wrist and knuckle epitopes, which indicates that the protein still remains bioactive to promote tissue restoration. This research forms the basis for understanding protein adsorption on patterned nanoscale graphite and can be extended to other substrate types.

## Figures and Tables

**Table 1:** Secondary structure content (%) of BMP-2 for monomers A and B at the beginning and after 20 ns simulations.

Secondary Structure	Initial Structure	Flat		LN1		LN2		CS1		CS2		SQ	
		A	B	A	B	A	B	A	B	A	B	A	B
α-helix	10.38	9.43	11.32	11.32	11.32	15.09	11.32	9.43	11.32	11.32	11.32	10.38	11.32
3_10_-helix	0	0	0	0	0	0	0	0	0	0	0	0	0
β-sheets	44.34	40.57	42.45	38.68	42.45	44.34	44.34	40.57	42.45	35.85	44.35	37.74	44.34

**Table 2:** Duration of SMD phase performed for each patterned substrate.

Type of Patterns	Timestep	Time (ps)
Flat	40,000	8
Linear grating 1 (LN1)	160,000	32
Linear grating 2 (LN2)	160,000	32
Circular slot 1 (CS1)	200,000	40
Circular slot 2 (CS2)	160,000	32
Square slot (SQ)	480,000	96

## Data Availability

The data presented in this study are available on request from the corresponding author.
